# Radiographic Comparison of Bovine Bone Substitute Alone Versus Bovine Bone Substitute and Simvastatin for Human Maxillary Sinus Augmentation

**Published:** 2018-01

**Authors:** Siamak Yaghobee, Amir Ali Reza Rasouli Ghahroudi, Afshin Khorsand, Sanaz Mahmoudi, Sahar Chokami Rafiei

**Affiliations:** 1 Associate Professor, Dental Research Center, Dentistry Research Institute, Tehran University of Medical Sciences, Tehran, Iran; Department of Periodontics, School of Dentistry, Tehran University of Medical Sciences, Tehran, Iran; 2 Pharmacist, Private Practice, Tehran, Iran; 3 Postgraduate Student, Department of Periodontics, School of Dentistry, Tehran University of Medical Sciences, Tehran, Iran

**Keywords:** Cone-Beam Computed Tomography, Sinus Floor Augmentation, Simvastatin

## Abstract

**Objectives:**

The aim of this study was to compare the efficacy of bovine bone substitute (Compact Bone B. ®) alone versus bovine bone substitute and simvastatin for human maxillary sinus augmentation.

**Materials and Methods:**

This study was conducted on 16 sinuses in eight patients. Radiographic assessments were done preoperatively (T0), immediately (T1) and at nine months after sinus grafting (T2). Alveolar bone height and density were assessed on cone beam computed tomography (CBCT) scans using Planmeca Romexis™ Imaging Software 2.2.

**Results:**

The change in alveolar bone height and density between T0, T1 and T2 was significant in both groups. Alveolar bone height (h0, h1, h2) and vertical height of the grafted bone (g1, g2) in three lines (anterior, middle and posterior) were not significantly different between groups. The grafted bone height shrinkage (%) in the anterior, middle and posterior limits of the augmented area were not significantly different between groups. The existing alveolar and grafted bone density increased significantly in both groups between T1 and T2, except for the existing alveolar bone density in the control group. There were no statistically significant differences between the alveolar bone density values obtained in TI and T2 between groups, except for the existing alveolar bone density at T1.

**Conclusions:**

This study did not show any significant positive effect for simvastatin in maxillary sinus augmentation based on radiographic examination.

## INTRODUCTION

Sinus pneumatization and alveolar ridge resorption decrease the amount of available bone in the posterior maxillary edentulous ridge and result in difficulties in dental implant placement [1–3]. The maxillary sinus augmentation by lateral wall approach has become a standard protocol to provide adequate bone quantity and quality to ensure placement of dental implants with sufficient length and acceptable primary stability [4–8]. Autogenous bone graft is the most predictable choice for augmentation surgeries even for extensive autogenous bone grafting.

Because of donor site morbidity, several artificial materials have been introduced such as allografts, xenografts and alloplasts hitherto [9, 10]. The results of bone augmentation with xenografts are the most favorable, and have been well documented in the literature [4]. Bone substitutes are resorbed over time and may consequently induce sinus repneumatization [11–14]. Bone changes can be assessed as change in bone height and density. Different radiographic modalities have been used for evaluation of grafted sinuses; however, panoramic radiography can hardly assess the maxillary sinus floor due to poor resolution [15]. Another modality is computed tomography (CT) which is used to assess the grafted sinus floor and to measure the height and volume of the available bone for implant placement [16]. Furthermore, magnetic resonance imaging may facilitate accurate assessment of the grafted sinus floor [17]. A reliable diagnostic method using a nasal endoscope has also been used to examine the maxillary sinus [18].

Nowadays, cone-beam computed tomography (CBCT) is considered as a great development in the field of dental radiology. CBCT can be used to assess the newly formed bone with high quality diagnostic images in three dimensions with lower patient radiation dose in comparison with other modalities and the conventional CT. In addition, no invasive procedure is needed [19–21].

Simvastatin, which is used orally to treat hypercholesterolemia and hyperlipidemia, has anti-resorptive actions and anabolic effects on bone. Up to now, the focus has been given to study the effects of locally administered simvastatin on bone formation [22, 23]. Previous studies assessed the application of simvastatin for treatment of fenestration defects in rats [24] and dogs [25], calvarial defects in rats [26], mandibular defects in rats [27], chronic periodontal defects and class II furcation defects in dogs [28]. These studies showed promising results for use of simvastatin for bone formation. Considering the above-mentioned facts, the aim of the present study was to radiographically evaluate the bone height and density 9 months after grafting of the maxillary sinuses with bovine bone substitute (Compact Bone B. ®, Dentegris International GmbH, Germany) alone versus bovine bone substitute and simvastatin.

## MATERIALS AND METHODS

Eight patients (16 sinuses) were selected among those presenting to the Periodontology Department of Dental Faculty of Tehran University of Medical Sciences requiring implants for the posterior maxilla and did not have sufficient amount of bone as determined on CBCT scans for dental implant placement. The patients were partially or completely edentulous in the posterior maxilla and all of them needed bilateral maxillary sinus augmentation. The exclusion criteria are listed in Table 1. The research protocol was reviewed and approved by the Ethics Committee of the Dental Research Center of Tehran University of Medical Sciences (IR.TUMS.REC.1395.2818). Before inclusion in the study, patients read, understood, and signed an informed consent form.

**Table 1.** Exclusion criteria

Patients requiring antibiotic prophylaxis for dental procedures
Any sinus pathology (acute or chronic) contraindicating sinus grafting
Patients with systemic diseases contraindicating oral surgeries
Patients who smoked more than 10 cigarettes daily
Women who were pregnant or wished to become pregnant during the period of the study
Patients under 18 years of age
Patients with diseases or use of medications known to affect bone metabolism, tissue regeneration and repair such as corticosteroids, bisphosphonates and etc.
Patients with a history of alcoholism or recreational drug abuse
Patients who had a history of cancer or radiation to the head and neck

### Surgical procedure:

Clinical photographs were taken before, during and after surgery. Procedures were performed under local anesthesia with different anesthetics. By reflection of a full thickness flap, the lateral wall of the sinus was exposed and osteotomy of the lateral sinus wall was performed with rotary burs. The wall and sinus membrane were elevated. Bovine bone substitute alone was placed in one side and bovine bone substitute with simvastatin was placed in the contralateral side. This study had a split mouth design. In this way, if one side was treated with bovine bone substitute, the other side was treated with bovine bone substitute and simvastatin or vice versa.

In the test sites, 0.5 cc of simvastatin solution (8 mg simvastatin in 0.5 cc of 70% ethanol) was mixed with 0.5 cc of bovine bone substitute. The same ratio of simvastatin to bovine bone substitute was used if additional material was required to fill the sinuses. A resorbable collagen membrane (SIC b-mem ®, invent Deutschland GmbH, Göttingen, Germany) was hydrated in sterile saline and placed over the lateral window. Primary flap closure was done with non-resorbable or resorbable sutures. A postoperative CBCT was taken to ensure that all graft materials were in place. Appropriate antibiotics, analgesics for 7–10 days and 0.2% chlorhexidine gluconate for two weeks, were prescribed. The patients were asked to return to the clinic 7–10 days after surgery for suture removal and postoperative assessment.

### Blinding:

Surgeons were not blinded to the materials applied to the sites. However, the examiner who performed the radiographic evaluations was blinded to the treatment and did not perform or supervise the surgery.

### Data analysis and evaluation techniques (radiographic):

CBCT was used for evaluation of sinus health, morphology and residual alveolar bone height and density. For all patients, radiographic assessments were done preoperatively (T0), immediately (T1) and at 9 months after sinus grafting (T2) using Alphard-3030 ® (Asahi Roentgen Ind. Co., Ltd., Kyoto, Japan). The scanning conditions were: tube voltage of 80 kV, tube current of 4 mA, time duration of 17 seconds and slice thickness of 1 mm. CBCT images were stored in DICOM format. The CBCT analysis was performed using Planmeca Romexis™ Imaging Software 2.2 ®. The dose of CBCT was approximately 25-1025 μSv. The common natural radiation was about 2.5 mSv/y, and the maximal radiation admissible was 50 mSv/y [29].

### Alveolar bone height analysis:

**Fig. 1:**
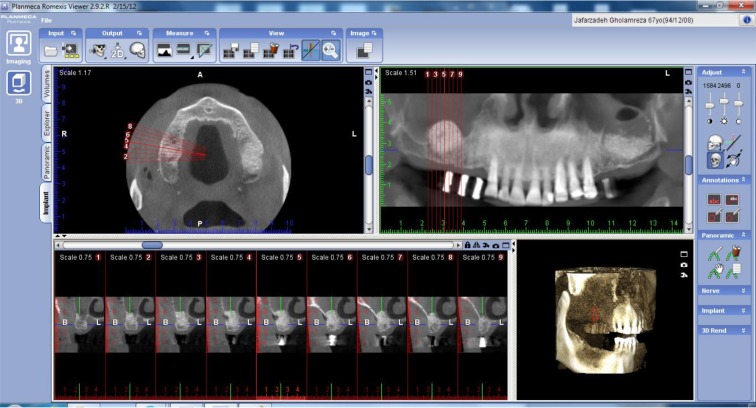
Alveolar bone height measurement using Planmeca Romexis™ Imaging Software 2.2®

Two lines which were the mesial and distal limits of the augmented area and a central point between these two lines were selected on CBCT scans.Alveolar bone height which was measured from the lowest point of the existing bone to the top of the grafted area (h0, h1, h2) and vertical height of the grafted bone (g1, g2) in these three lines (anterior, middle and posterior) were measured and compared with each other (Fig. 1).

### Bone density analysis:

**Fig. 2:**
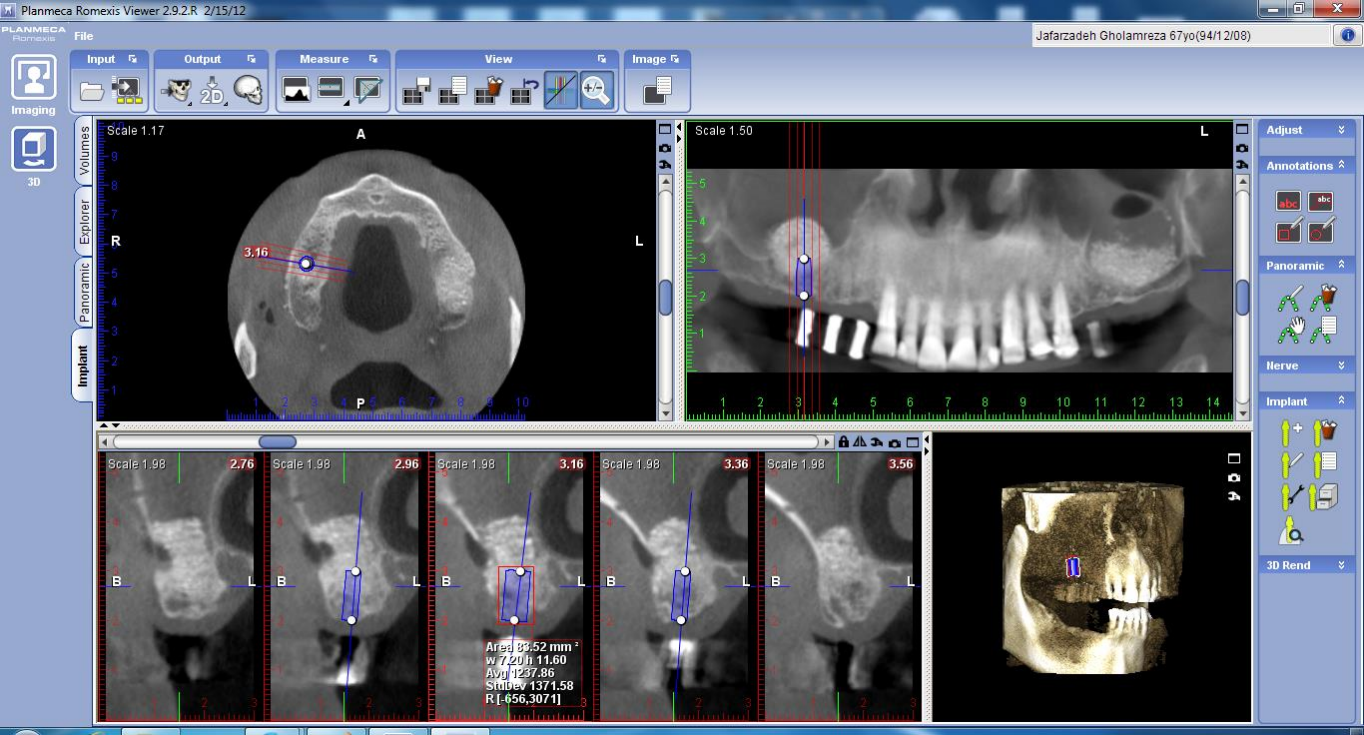
Alveolar bone density measurement using annotations option of Planmeca Romexis™ Imaging Software 2.2®

By using the “Add Implant” option of the software, an implant with proper size (4×10 mm) was chosen and placed over the grafted area on all scans. The bone density with approximately 1 mm thickness around the path of insertion of each implant was drawn by “Annotations- Measure Rectangle” option. Also, the implant was divided into two parts of 5 mm and then the bone density with approximately 1 mm thickness around each part was measured. The apical part was termed as the grafted bone and the coronal part was termed as the existing alveolar bone underneath the grafted region. The bone density of the sites was measured in Hounsfield units (HU) in all CBCT scans (Fig. 2).

### Statistical analysis:

For statistical analysis, SPSS version 22 was used. Data were normally distributed as checked by Kolmogorov-Smirnov test. Data were presented as mean± standard deviation (SD). Repeated measures ANOVA was used to compare the groups and time intervals; the parameters were compared between the test and control sites using paired sample t-test.

## RESULTS

The age of eight patients (six females and two males) ranged from 53 to 67 years with a mean age of 60.25±5.02 years. All patients healed without any complications.

### Bone height:

Table 2 show the summary of statistics for CBCT bone height at T0, T1 and T2 in the control (bovine bone substitute) and test (bovine bone substitute and simvastatin) groups. The alveolar bone height changes between T0, T1 and T2 were significant in both groups (P< 0.001). Alveolar bone height (h0, h1, h2) and vertical height of the grafted bone (g1, g2) in the three lines (anterior, middle and posterior) were not significantly different between the control and test groups (P>0.05). The grafted bone height shrinkage (%) in the anterior, middle and posterior limits of the augmented area was not significantly different between the control and test groups.

**Table 2.** Bone height (mm) evaluation with cone beam computed tomography. Test side (xenograft+ simvastatin), control side (xenograft only)

**Time**		**Preoperative**			**Immediate**						**9 months**						**Immediate/9 months**		

**Variable**		**Height (h0)**			**Total height (h1)**			**Height of graft material (g1)**			**Total height (h2)**			**Height of graft material (g2)**			**Shrinkage (%)**		

**Groups**		**Ant**	**Post**	**Mid**	**Ant**	**Post**	**Mid**	**Ant**	**Post**	**Mid**	**Ant**	**Post**	**Mid**	**Ant**	**Post**	**Mid**	**Ant**	**Post**	**Mid**
**Test**	**Mean**	4.38	3.75	4.08	15.81	15.75	16.81	11.44	12	12.74	14.66	14.60	14.70	10.26	10.85	10.63	10.31	9.16	16.57
**side**	**± SD**	0.45	0.54	0.80	1.78	1.50	1.43	1.62	1.73	1.38	1.87	1.14	1.92	1.65	1.16	2.03	5.20	5.02	12.55
**Contro**	**Mean**	3.90	3.94	3.98	15.96	17.68	17.84	12.06	13.58	13.66	14.36	15.80±	15.20	10.34	11.86	11.23	14.48	12.90	20.10
**l side**	**± SD**	0.76	0.77	1.03	1.46	1.76	2.18	1.80	2.17	2.57	1.88	2.08	2.86	1.94	2.43	3.33	6.87	7.84	12.64
**P value**		.102	.142	.015	.711	.971	.872	.569	.855	.495	.760	.632	.485	.741	.721	.863	.406	.091	.598

Table 3 shows the mean± SD values for the existing alveolar and grafted bone density in the control and test groups. The alveolar bone density changes between T0, T1 and T2 were significant in both groups (P<0.001). The alveolar and grafted bone density increased significantly in both groups between T1 and T2, except for the existing alveolar bone density in the control group which did not increase significantly (P=0.081).

**Table 3.** Bone density (HU) evaluation with cone beam computed tomography. Test side (xenograft+ simvastatin), control side (xenograft only)

**Time**		**Preoperative**	**Immediate**			**9 months**		

**Variables/ Groups**		**Total density(d0)**	**Total density(d1)**	**Grafted bone density**	**Existing alveolar bone density**	**Total density(d2)**	**Grafted bone density**	**Existing alveolar bone density**
**Test side**	**Mean± SD**	417.63± 191.23	975.75± 106.32	1022.50± 127.69	598.38± 150.16	1087.38± 115.91	1063.63± 83.50	662.88± 158.93
**Control side**	**Mean± SD**	438.13± 123.54	1019.88± 118.64	1103.50± 146.77	526.75± 90.76	1160.25± 125.24	1165.38± 114.06	563.75± 101.86
**Inter group comparison**	**P value**	.003	.804	.970	.013	.711	.996	.386

There were no statistically significant differences between the alveolar bone density values obtained at T1 and T2 in the two groups (P>0.05) except for the existing alveolar bone density at T1 that was significantly different between groups (P=0.013).

## DISCUSSION

The aim of the present study was to evaluate the bone height and density using CBCT scans at the site of either augmented maxillary sinus with bovine bone substitute or bovine bone substitute and simvastatin up to a period of nine months.

Inhibition of the enzyme 3-hydroxy-3-methylglutaryl coenzyme A reductase (HMGCoA reductase) and the following blockade of the mevalonate pathway is the most important mechanism of inhibition of bone resorption by simvastatin. Interference with the generation of isoprenoids that is another product of this pathway, results in disruption of vesicular fusion and ruffled border formation of osteoclasts, which are necessary for their bone-resorbing activity. It has been found that simvastatin increases the expression of bone morphogenetic protein-2, vascular endothelial growth factor, bone sialoprotein, osteocalcin, and type I collagen which result in upregulation of bone formation. Simvastatin increases osteoblast count, alkaline phosphatase activity and mineralization [30].

Healing time is an important factor for bone formation after sinus augmentation. As longer healing period provides a respite for more new bone to be formed, it can also prevent implant loading. Implant loading promotes osteogenesis and may act as a stabilizer for the maintenance of bone graft height [11]. With respect to these reasons, in our study, 9 months of healing period were allowed. However, Ozyuvaci et al. [31] compared a delayed procedure with implant placement in the second phase with immediate implant placement after sinus augmentation. After 6–8 months, no statistically significant difference was found in the vertical height reduction between the two groups.

In our study, bovine bone substitute was used for sinus augmentation. The fact that bovine bone resorbs slowly might be a negative factor, because it may prevent the new bone from reaching the surface of the implant after implant placement. In other words, the graft height may be well maintained over time, but it does not supply bone to contact the implant [11].

Valentini et al. [32] demonstrated new bone formation on the surface of a retrieved implant placed in Bio-Oss. In this study, CBCT analysis immediately after surgery showed that the bone height increased in the control and test groups. Between the two time points of immediately after surgery and 9 months after surgery, bone height decreased in the control and test groups without a statistically significant difference between them. The bone height at nine months was higher than the preoperative bone height but bone height decreased over time. Loss of vertical bone height has often been attributed to resorption of the graft material and repneumatization of the maxillary sinus which may be caused by positive intra sinus air pressure [33, 34].

Penetration of implant into the sinus membrane may increase the risk of infection [35]. Although, Branemark et al. [36] observed that penetration of implant into the sinus caused no unfavorable effects.

There was a statistically significant reduction in bone height between the two time points of immediately and 9 months after surgery in each group which was inconsistent with the results of Panagiotou et al. [1] who reported no difference between the alveolar bone height results obtained immediately and 8 months after augmentation. This may be justified by the application of different bone graft materials (Compact Bone B. ® vs Cera bone® and Bio-Oss®). These findings are consistent with those of Hallman et al. [37] who assessed dimensional changes following maxillary sinus floor augmentation with bovine hydroxyapatite and autogenous bone at 3, 12 and 24 months.

Also, these results are consistent with those of Hatano et al. [11]. In this study, graft height changes after maxillary sinus floor augmentation with a 2:1 autogenous bone/xenograft mixture and simultaneous placement of dental implants were evaluated radiographically. The overall height of bone graft decreased in the first 2-3 years after augmentation. After that, only minor changes occurred. But, graft height up to 96 months after augmentation was higher than that at baseline.

By comparing density 9 months after bone augmentation, we could not demonstrate positive effect of simvastatin as an adjunct (control group: 1160.25±125.24 HU and test group: 1087.38±115.91 HU). This bone density range was categorized as D2 according to the Misch category [38].

Despite the increase over time in the test group, these density values remained below the control group value. New bone density values were observed in favor of the control sides compared with test sides because the bone graft includes mineral while the test group received simvastatin to stimulate new bone formation with smaller amount of bone graft. This justification must be verified by histological analysis of bone core harvested from the site of implant placement. Thus, CBCT analysis may not be suitable for assessing the quality of available bone for implant placement. A range of bone density rather than absolute bone density value may a useful and accurate indicator of bone quality and primary stability of dental implants [39].

The mean bone density of the existing alveolar bone was 438.13±123.54 HU in the control side and 417.63±191.23 HU in the test side which was D3. These values were greater than those reported in some previous studies [40, 41] which may be attributed to the fact that previous studies were carried out on different populations. Immediately after surgery, bone density of the grafted site in the control side was 1019.88±118.64 HU; this value was 975.75±106.32 HU in the test side which was determined as D2. These values were greater than those reported by Froum et al, [4] who used mineralized cancellous bone allograft for maxillary sinus grafting. Presumably because bovine bone substitute (Compact Bone B. ®) appears very opaque on CBCT scans and thus changes over time, with regard to homogeneity, would be minimally visible.

Lima et al. [42] evaluated the influence of simvastatin and demineralized bovine bone matrix (DBBM) on repair of rat calvarial defects. The animals were divided into four groups: control without any filling; DBBM alone, DBBM with 2.2 mg/50 μL simvastatin and DBBM with 0.5 mg/50 μL simvastatin. The samples were X-rayed at day 30 and 60 and the radiographic density (gray levels) of the region of interest was calculated. X rays revealed that, on postoperative day 30, animals treated with a lower dose of simvastatin presented the lowest bone density; whereas, on postoperative day 60, the use of simvastatin, regardless of the dose, resulted in lower density than that observed in the control and DBBM group samples. Different results of this study may be due to different samples, defect type, carrier and higher dose of simvastatin.

Chauhan et al. [43] assessed the efficacy of simvastatin in mandibular third molar sockets over 3 months. Osseous regeneration was evaluated using standardized intraoral periapical radiographs. The study group received simvastatin (10 mg) powder along with gel foam as carrier moistened with 2 mL of saline solution. Significantly higher values were observed in the study group compared to the control group at days 1, 30 and 90. The positive result of this study may be due to different defect type, carrier and different dose of simvastatin.

In the present study, in all patients of both study groups, a barrier membrane was placed over the lateral window. A previous study stated that new bone formation in sinus grafts was greater, but not significantly, when either a non-absorbable membrane or a bio-absorbable membrane was used in comparison to grafted sinuses without a membrane [44].

Alveolar bone height and density analysis can assess the bone quality and quantity. But histological evaluation allows for the determination of the amount and nature of the newly formed bone, and the amount of connective tissue and residual graft materials. Thus, further studies are needed to assess the histological outcomes of simvastatin application for sinus augmentation.

## CONCLUSION

This study demonstrated no statistically significant positive effect of simvastatin for the maxillary sinus augmentation based on radiographic evaluations. Further studies are needed to assess the histological and radiological outcomes of simvastatin application with different doses or delivery forms for sinus augmentation.
